# Patterns of differences in wayfinding performance and correlations among abilities between persons with and without Down syndrome and typically developing children

**DOI:** 10.3389/fpsyg.2014.01446

**Published:** 2014-12-16

**Authors:** Megan Davis, Edward C. Merrill, Frances A. Conners, Beverly Roskos

**Affiliations:** Department of Psychology, The University of Alabama, Tuscaloosa, AL, USA

**Keywords:** down syndrome, route-learning, landmarks, spatial abilities, MA comparison

## Abstract

Down syndrome (DS) impacts several brain regions including the hippocampus and surrounding structures that have responsibility for important aspects of navigation and wayfinding. Hence it is reasonable to expect that DS may result in a reduced ability to engage in these skills. Two experiments are reported that evaluated route-learning of youth with DS, youth with intellectual disability (ID) and not DS, and typically developing (TD) children matched on mental age (MA). In both experiments, participants learned routes with eight choice point presented via computer. Several objects were placed along the route that could be used as landmarks. Participants navigated the route once with turn indicators pointing the way and then retraced the route without them. In Experiment 1 we found that the TD children and ID participants performed very similarly. They learned the route in the same number of attempts, committed the same number of errors while learning the route, and recalled approximately the same number of landmarks. The participants with DS performed significantly worse on both measures of navigation (attempts and errors) and also recalled significantly fewer landmarks. In Experiment 2, we attempted to reduce TD and ID vs DS differences by focusing participants’ attention on the landmarks. Half of the participants in each group were instructed to identify the landmarks as they passed them the first time. The participants with DS again committed more errors than the participants in the ID and TD groups in the navigation task. In addition, they recalled fewer landmarks. While landmark identification improved landmark memory for both groups, it did not have a significant impact on navigation. Participants with DS still performed more poorly than did the TD and ID participants. Of additional interest, we observed that the performance of persons with DS correlated with different ability measures than did the performance of the other groups. The results the two experiments point to a problem in navigation for persons with DS that exceeds expectations based solely on intellectual level.

## INTRODUCTION

Down syndrome (DS) is the most common genetic syndrome resulting in intellectual disability (ID; Dykens et al., 2000), with trisomy 21 being the most prevalent of several forms of DS. Due to a chromosomal abnormality involving an extra 21st chromosome, people born with this form of DS experience intellectual and learning impairments throughout their childhood and adult development. Accompanied by other health complications such as heart problems and hearing deficits, DS typically has a major impact on overall cognitive development. Individuals with DS generally present with moderate to severe ID. As a group, they commonly exhibit an IQ range of 25–55 and only rarely surpass a mental age (MA) of approximately 7 or 8 in adulthood (Pennington and Bennetto, 1998). Accordingly, children with DS generally acquire gross/motor and personal/social skills later than most typically developing (TD) children and struggle with the complex rules of language throughout their lives (National Down Syndrome Society, 2007). Further, DS often causes premature aging in middle adulthood that results in a further decline in IQ and even early onset Alzheimer’s (Wisniewski et al., 1985; Zigman et al., 1996). In the research reported here, we evaluated the performance of children and adolescents with DS on a complex cognitive skill known as wayfinding.

Wayfinding is a common spatial skill that refers to a general ability in which individuals navigate from one location to another unseen location via some mostly unmarked route. Descriptions of successful wayfinding commonly refer to at least two different aspects of wayfinding (Siegel and White, 1975; Golledge et al., 1995). Route-learning is a relatively simple method of wayfinding that can involve learning to travel along a specific path and making a specific sequence of movements along that path to reach the goal location. Reliance on survey knowledge is a more sophisticated method of wayfinding. It utilizes a more configural understanding of the interrelations among several routes in the environmental space, and can allow for the use of novel pathways to reach a goal location. In an unfamiliar environment, the learning of route and survey information depends on the understanding of spatial relations between places along that route and the ability to integrate overlapping information among several routes into a survey representation of the area (see Blades, 1997). Research on how children develop this basic understanding of their environments, represent spatial information about their environments, and use that information to navigate their environments indicates a fairly prolonged period of development, with dramatic increases in the efficiency of wayfinding skills occurring between the ages of 6 and 12 (Cohen and Schuepfer, 1980; Anooshian and Kromer, 1986; Allen et al., 1989; Cornell et al., 1989).

Our choice to focus on wayfinding skills of persons with DS was motivated by several separate considerations. One consideration is a line of research associated with the relatively common claim that persons with DS exhibit weak language skills and relatively strong visuo-spatial skills (Chapman and Hesketh, 2001; Moldavsky et al., 2001; Silverman, 2007; Davis, 2008 for reviews), which is often used to imply that spatial abilities are a strength in persons with DS (e.g., Wang, 1996). However, that claim appears to be an overgeneralization, in that the assertion of a relative strength in visuo-spatial ability in DS based on a small number of tasks. In fact, most of the evidence has been generated from performance on the Corsi block span task. A recent review of the relevant literature suggests a much more complex pattern of performance on various measures of spatial abilities (Yang et al., 2014). Yang et al. (2014) concluded that persons with DS exhibited an uneven pattern of performance across several spatial ability tasks and did not exhibit a particular strength relative to overall cognitive ability in any of them. Hence, a re-focusing of efforts to study spatial abilities in DS seems warranted.

Another consideration involves the growing body of evidence that implicates several different brain structures in the characterization of neurological deficits in DS. More specifically, structural MRI studies have indicated that total intracranial volume is smaller in those with DS than in those without DS, with the greatest differences being found in the cerebellum, brainstem, and frontal lobes (Kesslak et al., 1994; Raz et al., 1995; Aylward et al., 1999). Further, hippocampal volumes have been found to be smaller relative to TD individuals even after correcting for total brain volume (Pinter et al., 2001). While the hippocampus is involved in a wide range of cognitive activities that involve explicit learning processes, research has clearly demonstrated that the hippocampal and parahippocampal regions play an important role in spatial navigation (Burgess and O’Keefe, 1996; Burgess et al., 1998; Maguire et al., 1998; O’Keefe et al., 1999; Tripp, 2001). For example, Maguire et al. (1996) reported that PET scans of London taxi drivers indicate that the hippocampus shows increased activation when recalling routes around the city, but not when recalling famous landmarks. MRI investigations have shown that part of the hippocampus (the posterior) was enlarged in London taxi drivers and that the growth of the hippocampus occurred during their time as taxi drivers (Maguire et al., 2000). Lesion studies (Smith and Milner, 1981) have also reported that lesions to the hippocampus of humans result in impaired spatial memory and navigation.

A final consideration involves research evidence indicating that performance on small-scale spatial tasks does not necessarily predict performance on larger-scale spatial tasks (e.g., Hegarty et al., 2006). Hence, as pointed out by (Courbois et al., 2013), even if small-scale spatial abilities were to represent a strength in DS, it would still be necessary to evaluate their performance in large-scale spatial tasks such as wayfinding. In fact, Courbois et al. (2013) compared several aspects of wayfinding performance for a group of adolescents and young adults with DS to a group matched with them on chronological age (CA at approximately 22 years) and a group matched with them on MA (at approximately 7.7 years). Participants learned two short paths (two turns each) between two locations (A–B and A–C) in a virtual environment. Then they were asked to find the shortest route between the two locations that were not directly learned (B–C). The participants with DS took longer to learn the initial routes than did the equal CA-matched participants (and marginally longer than the MA-matched participants), remembered fewer landmarks along the route than did the CA- and MA-matched participants, and were less likely to find the short-cut than either comparison group. More recently, Purser et al. (2014) reported that the performance of persons with DS relative to TD children may depend on non-verbal ability, with participants with DS who were relatively higher on non-verbal ability performing similar to TD children and participants with relatively lower non-verbal ability performing more poorly than TD children.

Taken together, these results indicate that some aspect of wayfinding may be particularly problematic for at least some persons with DS and there is sufficient justification for continued research in this domain. In the present research, we focus on route-learning. Two experiments are presented here that were designed to address three basic questions about route-learning in DS. First, is route-learning a specific problem in persons with DS or do they exhibit difficulties similar to others with ID of mixed etiology? To address this question, in Experiment 1 we compared the performance of adolescents and young adults with DS to a group of participants with ID not resulting from DS with whom they were matched on MA. We also included a group of TD children matched on MA with both groups to assess whether the performance of the DS and mixed ID participants was below what might be expected based on their MA. Second, because landmark learning is a deficit in persons with DS (see Experiment 1 below and Courbois et al., 2013), can we reduce differences in route-learning performance between participants with and without DS by encouraging them to focus on landmarks during initial exposure to the route to be learned? In Experiment 2, we asked half of the participants in each group to identify landmarks as they were exposed to the route for the first time to determine if a focus on landmarks improved the performance of participants with DS more than the comparison groups. Third, can we identify factors that may be related to poor performance in route-learning? To address this question, we correlated other spatial and non-spatial abilities with route-learning performance to determine if route-learning was associated with similar basic abilities for the mixed ID and DS participants.

## EXPERIMENT 1

Experiment 1 compared route-learning in adolescents/young adults with DS, adolescents/young adults with mixed ID, and TD children all matched on MA. Participants were required to learn a route through a simple virtual environment. Along the path, several landmarks were placed at points where a turn decision was necessary or in the middle of a segment of the route. If participants with DS have difficulty with route-learning that exceeds expectations based on MA, we expected that they would take longer to learn the route and commit more errors during route-learning than would the participants with mixed ID and the TD children. In addition, memory for landmarks was assessed to determine if differences in landmark learning were associated with differences in route-learning.

### MATERIALS AND METHODS

#### Participants

Most of the participants of Experiment 1 were selected from individuals tested as part of a larger study on general learning abilities of persons with DS. They were recruited through local schools, local service centers, and a research participant registry established by one of the researchers. For the larger study, we have recruited 19 adolescents/young adults with DS (11 males and 8 females), 20 young adults with ID of mixed etiology (11 males and 9 females), and 10 TD children (five males and five females) thus far. Three participants with DS and two with mixed ID failed to complete the route-learning task and were excluded from further analysis in this study. The remaining groups consisted of 16 participants with DS (CA = 214.8 months, SD = 57.9; MA = 57.9 months, SD = 6.2), 18 participants with mixed ID (CA = 210.1 months, SD = 42.0; MA = 68.5 months, SD = 12.8), and 10 TD children (CA = 77.6 months, SD = 13.8; MA = 90.0 months, SD = 32.9). For Experiment 1, it was necessary to equate the three groups on MA. The groups with DS and mixed ID were equated by eliminating three participants with mixed ID whose MA was too high to match with the participants with DS and one participant with DS whose MA was too low to match with the participants with mixed ID. To obtain a matching TD group, we had to eliminate four of the original TD participants and add nine new TD participants with MA scores that were consistent with the participants with DS.

In the final grouping for Experiment 1, participants were 15 adolescents/young adults with DS (nine males and six females), 15 adolescents/young adults with ID of mixed etiology (eight males and seven females), and 15 TD children (eight males and seven females). Individuals with Williams syndrome were not included in the mixed ID group due to their known difficulties with navigation and spatial processing (e.g., Farran et al., 2012). The participants with DS had a mean CA of 217.9 months (SD = 60.8) and a mean non-verbal MA of 60.2 months (SD = 7.0). The participants with mixed ID had a mean CA of 212.9 months (SD = 51.1) and a mean non-verbal MA of 67.3 (SD = 11.1). The TD participants had a mean CA of 67.6 months (SD = 7.6) and a mean MA of 63.8 months (SD = 8.6). MAs were based on the Leiter International Performance Test—Revised (Leiter-R) Brief Form for the DS and ID participants. For the TD participants, MAs were derived from the Leiter-R Brief Form (six participants) or the matrices subtest of the Kaufman Brief Intelligence Test—Second Edition (KBIT-2; nine participants). Comparisons of the Leiter-R and KBIT-2 matrices have yielded similar means and a fairly strong correlation (*r* = 0.62) in children with autism and children with language impairments (Scattone et al., 2012). Hence, we assumed they would yield similar estimates of MA in our TD children. Etiologies of the Mixed ID group consisted of the following: eight unknown etiology, one fragile-X syndrome, two postnatal cause (malnutrition, TBI), one XXYY, one Rubinstein–Taybi syndrome, and two fetal alcohol spectrum disorder.

#### Design

Data reported for Experiment 1 were collected as part of the larger project or as a supplement to that project. The primary data were collected during a route-learning task (described below). The three groups (DS, ID, and TD) were compared on number of trials to learn the route, number of errors committed while learning the route, and number of landmarks recalled following their learning of the route.

#### Measures

***Leiter-R Brief Form.*** The Leiter-R is a standardized measure used to assess cognitive functioning in children and adolescents between the ages of 2 and 20. It is often used with individuals with ID because it is an entirely non-verbal measure of fluid reasoning and visualization. For the purposes of this study, only the Brief Form, which includes the Figure Ground, Form Completion, Sequential Order, and Repeated Patterns subtests, was used. These subtests measure visuo-spatial and inductive reasoning skills that are typically classified as fluid intelligence. The Leiter-R Brief Form correlates 0.85 with both the full version of the Leiter-R and WISC-III, its internal consistency reliability is between 0.88 and 0.90 depending on age, and its test–retest reliability is between 0.88 and 0.96 (Roid and Miller, 1997).

***Kaufman Brief Intelligence Test-2—matrices.*** Nine TD participants completed the matrices subtest of the KBIT-2. The matrices (non-verbal) subtest consists of 48 visual analogies presented on either 2 × 2 or 3 × 3 matrices with one element missing. The participants’ task is to choose the answer that best completes the pattern (no time limit). We used the KBIT-2 matrices subtest as an index of general non-verbal functioning for these participants in the TD group.

**FIGURE 1:**
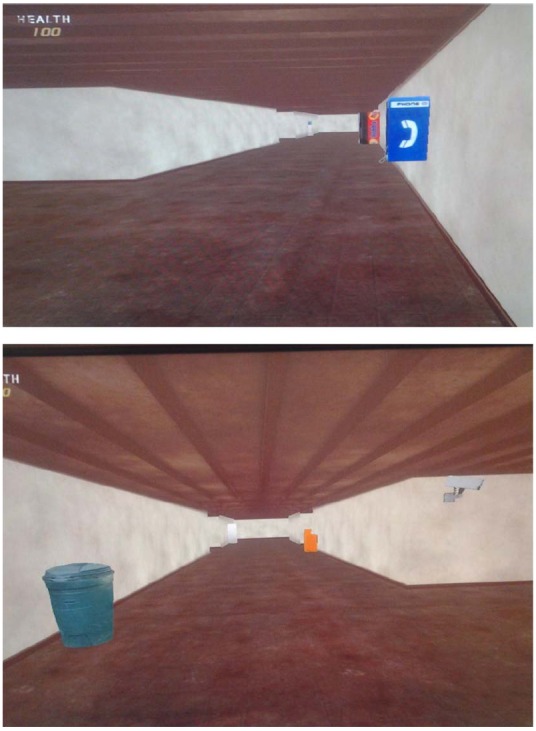
**Two segments of the route with landmarks (telephone and drink machine in upper screenshot and trash can and newspaper machine in lower screen shot)**.

***Route-learning task.*** Participants learned to navigate a route presented via a simple virtual environment that was constructed using the software program FPSCREATOR. Participants used the keyboard and mouse to navigate the maze. The mouse was used to change direction and the “W” key was used to go forward. Travel in reverse was not permitted and participants turned around in the maze to retrace their steps. Participants were trained in the use of the mouse and navigation key prior to the start of the route-learning task. In this task, participants were instructed to learn the shortest path to a specific target object. The virtual environment consisted of a set of hallways with eight choice points along the way. In order to maintain variability and participant interest, four of the choice points included two possible choices and four included three possible choices. Two of the choice points required participants to continue going straight, three required a right turn, and three required a left turn, with the locations of the turns being randomly determined. Sixteen landmarks were placed along the route, with half of the objects at choice points and the other half at non-choice points. Landmarks included objects such as vending machines, wall telephones, furniture, and pictures. Two sections of the route are presented in Figure 1. When completing the route-learning task, participants were first allowed to watch the experimenter travel the route once with directions and were then instructed to reproduce the correct path. The participants continued to navigate the path up to eight times until they were able to successfully navigate the environment without making any errors. Participants who were unable to successfully navigate the path without error were stopped after eight trials and received the maximum score of 8. For each participant, both the number of total trials needed to successfully navigate the route and number of errors, or wrong turns, was recorded. Further, in order to avoid participant frustration and limit time issues, prompts were given to prevent the participants from returning to a previous portion of the maze in which they had already successfully completed. Accordingly, each time a participant attempted to return to a choice point that had already been successfully navigated before, the participant was stopped, instructed that they had already been in that direction, and asked to choose another path. These attempts were also recorded as errors. Hence, total errors included turn errors plus number of prompts.

***Landmark recall.*** Upon completion of the route-learning task, participants were asked to recall as many of the landmarks as they could from the virtual environment. The number correct out of a possible 16 was recorded. Objects that were recalled were later identified by the experimenter as either being at a choice point or not at a choice point. Hence, the dependent measures were number of landmarks recalled at choice points and at non-choice points.

#### Procedure

Participants of the larger study completed the three tasks that are included in the present analysis (Leiter-R, route-learning, and landmark memory) as well as four other tasks. That are not included here. Two subtests of the Leiter-R Brief Form were always administered first (Figure Ground and Form Completion), a randomly selected task was administered second, and the last two subtests of the Leiter-R (Sequential Order and Repeated Patterns) were administered third. The remaining tasks were administered subsequently in random order, though the landmark recall always followed the route-learning task. Breaks were provided as needed after completion of each task. The full procedure took approximately 90 min to complete. The supplemental TD participants completed the matrices subtest of the KBIT–2, followed by the route-learning task and the landmark recall task. This took approximately 30 min.

### RESULTS

Primary data for Experiment 1 are presented in Table 1. A preliminary analysis of MA scores revealed that groups were not significantly different on measured MA [*F*(2,42) = 2.261, *p* = 0.117]. The analysis of route-learning was completed using multivariate analysis of variance procedures with Group as the independent variable and Number of Trials and Number of Errors treated as two dependent variables in the analysis. The MANOVA revealed a main effect of Group, *F*(4,82) = 3.778, *p* < 0.01, using Wilks’ lambda. The univariate analyses revealed a main effect of Group for Number of Trials [*F*(2,42) = 4.887, *p* = 0.012, ${\text{η}}_{\text{P}}^{\text{2}}$ = 0.189 and for Number of Errors [*F*(2,42) = 8.428, *p* = 0.001, ${\text{η}}_{\text{P}}^{\text{2}}$ = 0.286 Tukey HSD indicated that the participants with DS performed significantly worse on both measures than did either the ID or TD participants (all *p*s < 0.05). The ID and TD participants did not differ from each other. Because there were differences in MA, even though not significant, we conducted the analysis a second time using analysis of covariance procedures and the results were essentially unchanged; *F*(2,41) = 3.277, *p* = 0.048, ${\text{η}}_{\text{P}}^{\text{2}}$ = 0.138 for Number of Trials and *F*(2,41) = 6.436, *p* = 0.004, ${\text{η}}_{\text{P}}^{\text{2}}$ = 0.239 for Number of Errors.

**Table 1:** Descriptive data and dependent variable scores of Experiment 1 for each group and condition (SD in parentheses).

**Group**	**MA**	**Route-learning trials**	**Route-learning errors**	**Choice point landmarks**	**Non-choice point landmarks**
DS	60.2 (0.7)	7.0 (1.7)	32.7 (21.3)	2.1 (1.8)	0.9 (1.1)
Mixed ID	67.2 (11.2)	4.7 (3.0)	13.1 (13.1)	3.3 (1.0)	1.1 (0.9)
TD	63.8 (8.6)	4.6 (2.2)	12.7 (8.5)	3.6 (1.4)	1.1 (0.9)

The analysis of Landmark Recall was completed using a Group × Type of Landmark (Choice Point vs Non-choice Point) mixed design analysis of variance. There was a main effect of Group, *F*(2,42) = 3.279, *p* = 0.048, ${\text{η}}_{\text{P}}^{\text{2}}$ = 0.135, with the participants with DS recalling fewer landmarks overall than each of the other groups (*p*s < 0.05 using Tukey HSD). The main effect of Type of Landmark was also significant, *F*(1,42) = 74.976, *p* < 0.001, ${\text{η}}_{\text{P}}^{\text{2}}$ = 0.641, with landmarks at choice points being recalled more often than those at non-choice points. The interaction of Group × Type of Landmark approached significance, *F*(2,42) = 2.508, *p* = 0.094, ${\text{η}}_{\text{P}}^{\text{2}}$ = 0.107, indicating that the difference between groups was marginally more likely to be at choice point landmarks than not. However, this appears to be due to the participants with DS doing relatively poor in both conditions, rather than a pronounced improvement in the Choice Point conditions by the participants with ID and TD participants. A supplemental covariance analysis using MA as a covariate was again conducted and the results of the landmark analysis were also fundamentally unchanged with the effect of Group approaching significance, *F*(2,41) = 2.790, *p* = 0.090, and the Group × Type of Landmark being significant, *F*(2,4) = 3.562, *p* = 0.037. 

We evaluated one other aspect of our data. We conducted correlations between route-learning errors and total landmarks recalled for each group to assess the relation of landmark learning to route-learning. Note that a good performance in landmark learning results in a larger number than poor performance in landmark learning; the opposite is true for route-learning errors. Hence, a negative correlation reflects good performance on one associated with good performance on the other. The resulting correlation coefficients were *r* = –0.39 for the participants with DS, *r* = –0.59 for the participants with mixed ID, and *r* = –0.51 for the TD children. The first of these results approached significance (*p* = 0.075, one-tailed) and the last two were significant (*p* = 0.03 and *p* = 0.01, respectively, one-tailed). In each case, recall of more landmarks was associated with fewer errors in route-learning. Hence, it appears that landmark recall does share considerable variance with route-learning across groups.

## DISCUSSION

Experiment 1 confirmed our basic hypothesis. Adolescents and young adults with DS performed significantly worse on our measures of route-learning than did adolescents and young adults with mixed etiology ID and TD children. In contrast, participants with mixed ID performed at a level that was similar to TD children with whom they were matched on MA. Our results are therefore consistent with the observations of Courbois et al. (2013) who found that route-learning took longer for their participants with DS and extend those results to identify route-learning as a weakness in DS that is not simply due to their ID status. We think it is likely that the deficiency in route-learning exhibited by the participants with DS reflects the association between DS and impairments in the hippocampal regions of the brain (Aylward et al., 1999; Pinter et al., 2001; Pennington et al., 2003), as well as the role that the hippocampal regions play in spatial learning and navigation (e.g., Smith and Milner, 1981; Moser et al., 1993; Maguire et al., 1996).

We also found that our participants with DS recalled fewer landmarks viewed along the route that they traveled than did the ID and TD participants. This result is also consistent with Courbois et al. (2013). In our data, recall differences were associated more with landmarks at choice points relative to landmarks at less relevant locations. This difference in landmark recall between groups may be due to the well-established difficulties that persons with DS have with verbal learning (e.g., Chapman and Hesketh, 2001; Moldavsky et al., 2001; Silverman, 2007; Davis, 2008). Of course, the encoding and retrieval of explicit verbal episodic memories rely heavily on structures in the medial temporal lobe, including the hippocampus (Burgess et al., 2002). Hence, both features of navigation may be impacted by deficiencies in hippocampal functioning. Nevertheless, participants in all groups who were better able to recall landmarks were also the better route learners. Therefore, Experiment 2 focused on this issue more directly.

## EXPERIMENT 2

In Experiment 2, we were interested in whether or not instructions to focus on landmarks at choice points along the route would reduce the difference in route-learning performance between adolescent and young adults with DS relative to MA-matched participants with mixed ID and TD children. Research with children has generally found that identifying prominent landmarks along a path can enhance navigational performance (Cornell et al., 1989, 1992). For example, Cornell et al. (1989) examined the effects of landmark identification on navigational error in 6- and 12-year-old children. While some of the children were guided along a path with no instruction at all, others were told to look at either close or distal landmarks at points where changes in direction occurred. The results revealed that both age groups exhibited increases in navigational accuracy when instructed to look at specific landmarks, with the closer landmarks providing a more facilitative effect. Further, the 12-year-old children completed the task with fewer errors than the 6-year-olds (Cornell et al., 1989). Overall, these results indicate that the navigational accuracy of children is enhanced by the identification of prominent landmarks at choice points. Farran et al. (2010, 2012) have also reported that a similar relation between learning landmarks and route-learning can be found in TD children and children with Williams syndrome. Further, children with Williams syndrome can benefit from instruction that focuses their attention on relevant landmarks (see Farran et al., 2010, 2012).

In the present study, we evaluated the role of landmark memory on the route-learning performance of participants with DS, participants with mixed ID, and TD children. During their initial exposure to the route, half of the participants in each group were told to pay attention to the landmarks and identify them as they passed. If this manipulation successfully reduced the difference in landmark recall between the DS and other participants, then we might expect to see a corresponding reduction in the group differences in route-learning performance. One important methodological change was made from Experiment 1. We felt it was necessary to equate amount of exposure to the landmarks across individuals within groups to adequately assess landmark memory. Therefore, participants only reproduced the route once following their initial exposure to the path. Hence, their route-learning performance in Experiment 2 was based on errors made during this single attempt to reproduce the route.

### MATERIALS AND METHODS

#### Participants

A total of 59 participants were recruited for this study including 20 individuals with DS, 18 individuals with mixed-etiology ID not resulting from Williams syndrome, and 21 TD children. Four participants, one TD and three DS, did not complete the study due to either a lack of desire to complete all of the tasks or an inability to meet the requirements of the tasks. Four additional TD children were excluded because their assessed MAs were too high for matching purposes. Two participants with ID, one with DS and one without DS were excluded to facilitate MA matching. For the final sample, there were 16 participants in each group. The participants with DS (12 males and 4 females) had a mean CA of 223 months (SD = 52.2) and a mean MA of 65.1 months (SD = 18.9). The participants with mixed ID (eight males and eight females) had a mean CA of 241.0 months (SD = 14.4) and a mean MA of 71.2 months (SD = 17.1). The TD participants (eight males and eight females) had a mean CA of 93 months (SD = 8.2) and a mean MA of 73.9 months (SD = 14.3). The DS and mixed ID participants were between the ages of 12 and 25 and were recruited from local service organizations. Children in the TD comparison group were recruited from the local schools. Etiologies of the Mixed ID group as reported by parent/guardian consisted of the following: five unknown etiology, three unreported by parent/guardian, two fragile-X syndrome, two postnatal cause (malnutrition, TBI), two comorbid with cerebral palsy, and two fetal alcohol spectrum disorder.

#### Design

The independent variables were participant Group (DS, ID, or TD) and Landmark Identification (identify or no instruction to identify). The dependent variables were number of errors in route-learning and number of landmarks recalled.

#### Measures

***Kaufman Brief Intelligence Test-2.*** All participants completed the matrices subtest of the KBIT-2.

***Route-learning task.*** The route participants learned was similar to the one used in Experiment 1. There were two fundamental differences in the procedure of Experiment 2. One difference was that during initial exposure to the layout, correct paths were designated by a green light at the choice points and incorrect paths were designated by red lights. During their initial exposure to the route, participants actively navigated the maze by following green lights that indicated the correct path and ignoring paths designated by red lights, which indicated wrong turns. All participants were instructed to concentrate while traveling through the maze because the lights would be removed in the next part of the task. The second difference was that following the self-navigation exposure trial, participants were given only one trial to reproduce the route on their own without the assistance of the green and red lights. The number of wrong turns made by participants while navigating the route was recorded. As in Experiment 1, in order to avoid participant frustration and time issues, prompts were given to prevent the participants from returning to a previous portion of the maze in which they had already successfully completed. Accordingly, each time a participant attempted to return to a choice point that had already been navigated successfully the participant was stopped, instructed that they had already been in that direction, and asked to choose another path. Each time a prompt was required, a route-learning error was recorded. Thus, number of errors in route-learning was computed by adding the number of wrong turns and the number prompts required. Number of errors made during the reproduction attempt was the dependent measure for route-learning.

***Landmark recall.*** Following their reproduction of the route, participants were asked to recall as many objects as they could that were seen along the path. Number of landmarks recalled was the dependent measure.

***Procedure.*** All participants were first administered the non-verbal matrices subtest of the KBIT-2. Half of the participants from each group were then randomly assigned to each of the two experimental conditions for completion of the virtual route-learning task. In both conditions, participants were exposed to the initial learning phase that introduced them to the virtual environment. Participants in the landmark instruction condition were told to pay special attention to the landmarks because they may be helpful in navigating the maze. The participants in this condition were also asked to identify the landmarks in the environment as they passed them by saying them aloud to the experimenter. All responses were recorded for accuracy of identification. The participants had no difficulty identifying the objects. Participants in the no instruction condition were only given the directions on following the green lights and were not asked to identify any of the landmarks. Following the self-navigation exposure trial, participants were asked to reproduce the route on their own without the assistance of the green and red lights. The number of wrong turns made by participants while navigating the route was recorded. Upon completion of the route-learning task, participants were then asked to recall as many of the landmarks as they could from the virtual environment. The entire procedure took approximately 30 min to complete.

### RESULTS

Primary data for Experiment 2 are presented in Table 2. A preliminary analysis of MA scores revealed that groups were not significantly different on measured MA [*F*(2,45) = 1.185, *p* = 0.315]. The analysis of route-learning was done using analysis of variance procedures with Group and Landmark Identification as the independent variables and Number of Errors in route-learning treated as the dependent variable in the analysis. The analysis revealed a main effect of Group, *F*(2,45) = 6.23, *p* < 0.01, ${\text{η}}_{\text{P}}^{\text{2}}$ = 0.217. Participants with DS committed more errors than either the participants with ID or the TD children (6.82 vs, 3.28 vs. 4.28, both *p*s < 0.05 using Tukey HSD). The participants with mixed ID and the TD participants did not differ. There was also a main effect of Landmark Identification, *F*(1,45) = 4.674, *p* < 0.05, ${\text{η}}_{\text{P}}^{\text{2}}$ = 0.094. Participants who identified landmarks during exposure committed fewer errors during route-learning performance than those who did not. This is clearly the case for at least the participants with DS and the TD children, although not necessarily for the participants with mixed ID who performed very well in the no instruction condition. However, the interaction of these variables was not significant, suggesting that the effect of Landmark Identification did not significantly differ for the three groups. Supplemental covariance analyses using MA as a covariate confirmed these results; *F*(2,45) = 5.53, *p* < 0.01 for group, and *F*(1,45) = 4.39, *p* < 0.05 for Landmark Identification.

**Table 2:** Descriptive data and dependent variable scores of Experiment 2 for each group and condition (SD in parentheses).

**Group**	**MA**	**Identify instructions**	**Route-learning errors**	**Landmarks recalled**
DS	65.1 (18.9)	Yes	5.9 (3.8)	4.1 (2.1)
		No	7.8 (2.7)	1.1 (1.4)
Mixed ID	71.2 (17.1)	Yes	3.3 (2.7)	6.1 (0.6)
		No	3.2 (2.1)	2.7 (2.5)
TD	73.9 (14.3)	Yes	3.0 (2.4)	4.4 (1.3)
		No	6.6 (3.9)	2.6 (1.6)

The analysis of landmark recall yielded a significant main effect of Group, *F*(2,45) = 4.703, *p* < 0.05, ${\text{η}}_{\text{P}}^{\text{2}}$ = 0.173 and a significant main effect of Landmark Identification, *F*(1,45) = 30.407, *p* < 0.01, ${\text{η}}_{\text{P}}^{\text{2}}$ = 0.403. The Group × Landmark Identification interaction was not significant. Tests of simple effects indicated that the participants with DS remembered significantly fewer landmarks than the participants with mixed ID (*p* < 0.01 using Tukey HSD), with the TD participants not being significantly different from either group. Hence, the deficiency exhibited by the DS participants in landmark recall was not as reliable as the deficiency exhibited by this group in route-learning. While it appeared that landmark identification did benefit the route-learning performance of the DS participants and TD children, it did not significantly impact the magnitude of the group differences in route-learning among our groups of participants. Further, the correlation between route-learning errors and landmark recall for our sample was a moderate –0.46, as it was in Experiment 1. However, only the participants with DS (–0.64) and with mixed ID (–0.45) exhibited significant correlation coefficients. Landmark learning was not significantly related to route-learning performance of the TD children (*r* = –0.15). Still, these data indicate that several additional factors need to be taken into account to explain the poor route-learning performance of persons with DS. More specifically, landmark learning per se did not fully explain the observed group differences in route-learning performance. Again, supplemental covariance analyses using MA as a covariate confirmed these results; *F*(2,45) = 3.33, *p* < 0.05 for group, and *F*(1,45) = 43.70, *p* < 0.01 for Landmark Identification.

### DISCUSSION

The basic results of Experiment 2 corroborated and extended the results of Experiment 1. Adolescents/young adults with DS performed significantly worse on a measure of route-learning than did participants with ID and TD children. However, the group differences were not as consistent, overall, in Experiment 2 as they were in Experiment 1. For example, the TD children without instructions to identify landmarks were more like the participants with DS than the participants with mixed ID, even though the interaction was not significant. We suspect that our change in procedures accounted for this difference. In Experiment 1, participants were given multiple trials to learn the route whereas in Experiment 2 they only reproduced the route once after initial exposure to it. The procedural change was necessary to control for exposure to the landmarks in Experiment 2 to evaluate the landmark identification manipulation. Because the problems exhibited by persons with DS in route-learning are likely to reflect learning problems, it is reasonable to expect that group differences will be greater when the participants are given more time to learn the route. Over time, the participants with DS would likely fall further and further behind as they fail to keep up with the any learning exhibited by participants with mixed ID and TD children. This may also help to explain why the participants with mixed ID performed better (although not significantly) than the TD children. The learning advantage of the TD children may well appear with more opportunities to learn the route.

Also similar across Experiments 1 and 2 was the observation that landmark recall was especially difficult for participants with DS. They recalled fewer landmarks, overall, than did the participants with mixed ID. However, they did not differ significantly in recall from the TD children. As noted earlier, it is very possible that this is another manifestation of the hippocampal deficiencies exhibited by persons with DS (e.g., Chapman and Hesketh, 2001; Moldavsky et al., 2001; Silverman, 2007; Davis, 2008). Explicit episodic memory also relies on hippocampal structures to be successfully encoded and retrieved.

## RELATION OF ROUTE-LEARNING SKILLS TO OTHER COGNITIVE PROCESSES: SUPPLEMENTAL ANALYSES

While the previous experiments indicated a group differences in performance between persons with and with DS in route-learning,**** we were not able to identify how the participants with DS processed route information differently from participants without DS. We conducted several supplemental analyses to address that issue. More specifically, as part of the larger study from which the data reported in Experiment 1 were drawn, two other tasks were included to identify basic abilities that may be related to route-learning performance in persons with and without DS. One task was an explicit verbal learning task (Word Learning) and the other was an implicit spatial memory task (Contextual Cueing).

Committing a list of words to memory was considered a standard measure of explicit verbal episodic learning and was expected to be related to route-learning performance of participants without DS. Verbal explicit memory was selected as a plausible correlate of route-learning because it likely underlies landmark recognition, remembering what action takes place at each landmark, and remembering the order of landmarks and actions. With adequate verbal memory skills, we expect that landmarks and turns can be explicitly combined and remembered. Hence, we would expect that word learning would be associated with route-learning performance of the participants with mixed ID. However, because of their poor verbal skills, it may be that word learning would be less associated with the route-learning performance of the participants with DS if they attempt to use non-verbal rather than their relatively weaker verbal skills to perform the task.

Contextual cueing generally involves an attentional guidance mechanism that makes use of previously experienced visuo-spatial regularities in the environment to facilitate search for a target object. Facilitation effects develop without intention and awareness and are assumed to reflect a form of implicit spatial learning (Chun and Jiang, 1998). In a typical contextual cueing task, participants are presented displays of multiple stimuli and are required to search for a particular target. The relative location of the target in some displays can be predicted by the positions of the other stimuli in the display, which remain the same over repeated trials during the procedure. After many repetitions, learning in this task is evidenced by faster response times on displays where the target location can be predicted from the other stimuli relative to displays in which the target cannot be predicted (Chun and Jiang, 1998). We have previously shown that persons with ID exhibit contextual cueing effects (Merrill et al., 2014). Hence, implicit spatial learning was selected as a plausible alternative method of performing route-learning by persons with DS. If persons with DS have difficulty with explicit more than implicit forms of spatial learning, then they may use implicit spatial learning processes to facilitate route-learning. If they do, this may result in a stronger correlation between contextual cueing and route-learning for participants with DS relative to participants with mixed ID.

### MATERIALS AND METHODS

#### Participants

The participants included in these analyses were all 16 participants with DS (CA = 214.8 months, SD = 57.9; MA = 57.9 months, SD = 6.2) and 18 participants with mixed ID (CA = 210.1 months, SD = 42.0; MA = 68.5 months, SD = 12.8) who completed the route-learning task as part of the larger study described in Experiment 1. The difference between groups in MA using the full sample was significant (*p* < 0.01), but was not considered relevant because we were not comparing mean performance between groups.

#### New measures

***Word learning.*** This task measures verbal learning and memory over repeated presentations and was patterned after the modified NEPSY list learning task used by Pennington et al. (2003). Participants were orally presented a list of 15 words by the researcher. After the presentation of the entire list, participants were asked to recall as many words as possible. The list, followed by immediate recall, was repeated five times. Following Pennington et al. (2003), we used the total number of words recalled across the five learning trials as our measure of episodic/explicit verbal learning.

***Contextual cueing.*** This task was patterned after Merrill et al. (2013).**** Participants were presented displays of multiple cartoon characters on a computer screen (five different characters appearing four times each and one character appearing once). They were asked to locate the character that appeared only once (Jiminy Cricket) as rapidly as possible. During the acquisition phase, participants received 4 blocks of 24 trials (a total of 24 presentations of each of four predictable displays). The general location of Jiminy Cricket (upper left, upper right, lower left, and lower right quadrant) could be predicted by the location of the other characters in the displays. Participants responded by touching the quadrant in which the target appeared on a touch screen monitor. Response times were recorded to the nearest ms. Each trial began with a fixation cross for 750 ms and ended when the participant made a response. In the test phase that immediately followed the acquisition phase, participants received 48 trials, 24 trials using the same predictable acquisition displays and 24 using new unpredictable displays. The main measure of implicit contextual learning was the degree of facilitation exhibited for predictable relative to unpredictable displays. This was calculated as a proportion of facilitation for predictable relative to unpredictable displays using the formula:

[RT (Unpredictable Displays) – RT (Predictable Displays)]/RT (Unpredictable Displays). Larger facilitation effects associated with contextual cueing would thus produce larger positive scores.

### RESULTS AND DISCUSSION

The primary data used in the correlations are presented in Table 3. Correlations were conducted separately for the participants with DS and participants with mixed ID. We initially conducted a correlation between contextual cueing and word learning for each group. The resulting correlation coefficients were *r* = 0.07 for the participants with DS and *r* = –0.25 for the participants with mixed ID. Neither individual correlation nor the difference between correlations was significant. Next we conducted correlations between these two abilities and route-learning errors for each group. Note that good performance in word learning and contextual cueing are associated with larger numbers whereas good performance in route-learning is a smaller number. Hence, negative correlations indicate that good performance on one is associated with good performance on the other. The correlations between route-learning errors and word list learning were *r* = 0.27 for the participants with DS and *r* = –0.57 for the participants with mixed ID. This difference between correlation coefficients was significant (*p* = 0.015). Correlations between route-learning errors and contextual cueing were *r* = 0.43 for the participants with DS and *r* = –0.25 for the participants with mixed ID. The difference between these correlation coefficients approached significance (*p* = 0.054). Because these correlations were significantly different for the participants with DS and participants with mixed ID, it seems clear that their performance on the route-learning task was attributable to very different learning mechanisms or styles.

**Table 3:** Means for route-learning performance and cognitive ability measures for supplemental analyses (SD in parentheses).

**Group**	**Route-learning errors**	**Words recalled**	**Contextual cueing proportion of facilitation**
DS	32.3 (19.9)	21.2 (8.7)	0.06 (0.11)
Mixed ID	10.7 (11.6)	31.4 (8.6)	0.04 (0.10)

We look first at the correlations between word learning and route-learning errors. The strongest correlation we observed was between word learning and route-learning errors of persons with mixed ID. More words learned were associated with fewer route-learning errors for this group. This is as expected because learning landmarks and learning how to turn at each landmark would likely rely heavily on verbal learning strategies that may be reflected in the word learning task. In contrast, the participants with DS actually exhibited a weak positive correlation between these variables indicating that higher scores in word learning were actually associated with more route-learning errors. It is not clear why this would be true. Perhaps the participants with DS were able to use some abilities other than explicit verbal memory to facilitate route-learning performance. Alternatively, it may be that participants with DS who were higher in verbal scores tried to use their abilities to facilitate route-learning but selected an approach that was less successful. Unfortunately, our data do not shed light on what that ability may be. Nevertheless, this may explain why instructions to identify landmarks did not have a significant impact on differences in route-learning performance between persons with DS and those with mixed ID.

Turing to the correlations between contextual cueing and route-learning errors, the positive correlation between route-learning and contextual cueing for the participants with DS indicated that participants who exhibited the largest contextual cueing effects committed the most errors in route-learning. Because contextual cueing reflects a relatively implicit attentional guidance effect, it seems that the participants with DS may be implicitly learning something about the environment that actually impedes their route-learning. Perhaps they are drawn to some particular landmarks or locations because of their appearance and not because they provide useful information about where to turn. If they are unable to inhibit these pre-potent tendencies, then difficulties in route-learning may result. In contrast, the small negative correlation between contextual cueing and route-learning errors for the participants with mixed ID suggest that implicit spatial learning is not very closely related to route-learning performance in this group. Hence, it seems reasonable to conclude that these participants were not influenced by implicit associations made between landmarks along the route either because they did not learn them or were able to inhibit their impact if they did.

While these differences in correlations are intriguing and suggest avenues of future research, it is necessary to evaluate them with some caution. They are based on relatively small samples. As a result, they are subject to the influence of outliers and a lack of statistical power. Nevertheless, they do indicate that our participants with and without DS are somewhat different in their approach to performing our route-learning task due to either choice or necessity.

## GENERAL DISCUSSION

Across two experiments, we observed that adolescents and young adults with DS exhibit serious difficulties with route-learning. The problems they exhibit exceed expectations based on their overall non-verbal ability relative to both TD children and persons with ID resulting from etiologies other than DS. Relative to both comparison groups, the participants with DS took twice as many trials to learn the route, made twice as many errors reproducing the path to the target during learning, and learned significantly fewer landmarks along the route. We think it is likely that this deficiency in route-learning exhibited by the participants with DS reflects, at least in part, the association between DS and impairments in the hippocampal regions of the brain (Aylward et al., 1999; Pinter et al., 2001; Pennington et al., 2003) and the role that the hippocampal regions play in spatial learning and navigation (e.g., Smith and Milner, 1981; Moser et al., 1993; Maguire et al., 1996). Courbois et al. (2013) also reported a difference in number of trials needed to reach criterion in route-learning performance, although they found that most of their participants with DS learned their relatively shorter routes. We are somewhat less confident that our participants would learn routes on their own, especially in more real world conditions. More specifically, it is highly unlikely that individuals will traverse the exact same route 8–10 times in a row until they could find their way without error. While this could be implemented as a training strategy, typically the amount of time in between instances of traveling the same route may be quite large (e.g., an entire day or longer) and thereby introduce even more difficulties for the person with DS.

In addition to general route-learning difficulties, our participants with DS remembered fewer landmarks that they passed along the way. In Experiment 1, the participants with DS actually saw the landmarks twice as often before recall (it took more trials for them to learn the route) and still remembered only half as many of the landmarks. This may also be related to hippocampal function because the hippocampus is required to produce successful verbal episodic memories (e.g., Hirshhorn et al., 2011; Nadel and Peterson, 2013). In Experiment 2, we assessed whether having participants focus on landmarks might reduce the difficulties in route-learning experienced by persons with DS. This is a common instruction when trying to teach children to learn a route (Cornell et al., 1989). While learning landmarks did reduce the number of errors made in the route-learning task for the participants with DS, it did so for all of our participants and did not allow the participants with DS to perform closer to the level of the mixed ID participants and the TD children. In fact, when all participants were asked to identify the landmarks as they passed them, there was still a significant difference in landmark memory among groups. Perhaps a more extensive approach to learning landmarks would benefit the participants with DS enough to reduce their route-learning difficulties. But, it is clear that simply identifying landmarks along the route is not a quick fix for the problem.

Interestingly, when we compared route-learning performance to two other targeted abilities, verbal episodic memory and implicit spatial memory, we found a very different pattern of correlations between these abilities and route-learning for the participants with DS and the participants with mixed ID. It seems clear that the participants with mixed ID were successfully engaging explicit episodic memory processes to facilitate route-learning performance. Basic verbal learning was associated with route-learning for the mixed ID participants (supplemental analyses) to roughly the same degree that landmark recall was associated with route-learning (Experiment 1). This was not what we observed for the participants with DS. While better landmark recall was moderately correlated with fewer route-learning errors, higher verbal memory scores were actually associated with more route-learning errors (although not significantly so) for the participants with DS. Hence, it would seem that some other variable was actually interfering with the ability of persons with DS to engage in landmark learning and use that to facilitate route-learning. One candidate for producing interference may be implicit spatial associative learning as measured by contextual cueing. Contextual cueing is generally considered a benefit because it guides attention to useful locations in the environment based on the implicit learning of the spatial layout (e.g., Goujon et al., 2009). However, it is also plausible that contextual cueing can guide attention to the wrong location (because participants initially target landmarks that are more attractive or interesting). Once learned, it may be difficult to readjust attention based on a newer understanding of the environment or a change in goals (see, for example, Makovski and Jiang, 2010). Hence, an inability to inhibit an implicit attentional guidance system could potentially interfere with route-learning for persons with DS.

Because of their poor verbal skills, it may be difficult to effect improvement in route-learning of persons with DS by training verbally based strategies. Hence, the result with the most immediate possibility for modifying their route-learning performance may be to focus on the interference to route-learning that seems associated with implicit spatial learning. In this case, we would not need to train a new learning approach. Rather, we would only need to refocus learning. Although limited to learning individual routes one at a time, it may be possible to present a series of pictures along a given route that enhance attention to relevant landmarks and devalue irrelevant landmarks. For example, individuals could search for a target that depicts the goal of the route-learning task placed next to a relevant landmark in the scene. If the relevant landmarks subsequently are the ones that elicit first attention, it is possible that individuals may learn paths associated with those landmarks more easily. Importantly, contextual cueing effects are easily established and can be learned within 5–10 simple exposures even in young children (Dixon et al., 2010). Further, we know that contextual cueing of scenes that approximate real environments have been demonstrated (Chun and Nakayama, 2000; Brockmole and Henderson, 2006; Goujon, 2011). Hence, we expect that persons with DS can also exhibit contextual cueing effects under these conditions as well. The challenge will be programming a contextual cueing procedure to produce a particular result.

Several limitations of the present research should also be mentioned. One of the clear limitations of this research is that our results were obtained in a virtual environment. While there is good evidence to suggest that observations made in virtual environments are often very similar to those obtained in real environments (see Koenig et al., 2009), it is not certain that this general rule applies to all conditions and participants. It will be necessary to establish that persons with DS perform similarly in virtual and real environments to validate results such as those we obtained. A second limitation is associated with how we introduced the route-learning task. We only allowed participants to view or navigate the route with directions once before they attempted the route on their own. Further, all of the participants introduced to the route in exactly the same way. Our results suggest that individuals with DS may not use the same basic abilities as our participants without DS to learn routes. We may have inadvertently produced a procedure that put the participants with DS at a disadvantage if our route and procedure favored persons with stronger verbal skills. Additional research using several environmental variations may prove valuable in assessing the generalizability of the performance differences between groups that we observed. This approach would also assist in identifying any particular skills that persons with DS may be able to effectively use in the activity of route-learning.

## CONCLUSION

Persons with DS experience difficulties with route-learning that are much greater than would be expected on the basis of their non-verbal MA. The participants with DS committed more errors than the participants with mixed ID and TD participants in a navigation task in two experiments. In addition, they recalled fewer landmarks in both experiments. Of primary interest was the fact that correlations between number of route-learning errors and two basic cognitive abilities, verbal episodic memory and implicit spatial learning, revealed a different pattern of correlations for the participants with DS and the participants with mixed ID. This result suggests that the participants with and without DS may have engaged different processing abilities to perform the route-learning task. In the future, we hope to be able to identify what those information processing differences may be and to use that information to develop appropriate training procedures for persons with DS.

### Conflict of Interest Statement

The authors declare that the research was conducted in the absence of any commercial or financial relationships that could be construed as a potential conflict of interest.
